# Comparison among TonoVet, TonoVet Plus, Tono-Pen Avia Vet, and Kowa HA-2 portable tonometers for measuring intraocular pressure in dogs

**DOI:** 10.14202/vetworld.2021.2444-2451

**Published:** 2021-09-21

**Authors:** João Victor Goulart Consoni Passareli, Felipe Franco Nascimento, Giovana José Garcia Estanho, Claudia Lizandra Ricci, Glaucia Prada Kanashiro, Rogério Giuffrida, Silvia Franco Andrade

**Affiliations:** 1 Postgraduate Program in Animal Science, UNOESTE, Presidente Prudente, São Paulo, Brazil; 2 Department of Veterinary Ophthalmology, Veterinary Hospital, UNOESTE, Presidente Prudente, São Paulo, Brazil.

**Keywords:** applanation tonometry, direct manometry, dogs, Goldmann tonometry, rebound tonometry

## Abstract

**Background and Aim:**

Tonometers are an important instrument for measuring intraocular pressure (IOP) in the diagnosis of glaucoma or uveitis. This study aimed to compare the accuracy of the main types of tonometers with different IOP measurement methodologies in dogs: TonoVet and TonoVet Plus (rebound), Tono-Pen Avia Vet (applanation), and Kowa HA-2 (Goldmann applanation).

**Materials and Methods:**

IOP was measured in 152 eyes of 76 dogs. A postmortem study was performed by comparing manometry and tonometry values and calculating the correlation coefficient (r^2^), *in vivo* real IOP (manometry) among the tonometers was compared, and an outpatient study was conducted with healthy eyes and eyes with signs of glaucoma and uveitis.

**Results:**

In the postmortem study, the values of r^2^ in descending order were Kowa (0.989), TonoVet Plus (0.984), TonoVet (0.981), and Tono-Pen Avia Vet (0.847). The IOP values in mmHg in the *in vivo* study were as follows: Aneroid manometer (16.8±2.5.7), TonoVet (18.1±2.9), TonoVet Plus (20.6±2.3), Tono-Pen Avia Vet (17.1±2.5), and Kowa (16.1±1.7); in outpatient clinics: TonoVet (16.8±3.8), TonoVet Plus (19.2±2.9), Tono-Pen Avia Vet (16.2±2.4), and Kowa (15.0±1.3); glaucoma: TonoVet (30.2±3.5), TonoVet Plus (35.0±6.1), Tono-Pen Avia Vet (29.5±4.2), and Kowa (23.9±5.0); and uveitis: TonoVet (14.2±1.4), TonoVet Plus (17.6±1.9), Tono-Pen Avia Vet (13.7±2.1), and Kowa (12.6±1.7)**.**

**Conclusion:**

There was a strong correlation between IOP values and manometry in all the tonometers. The highest values were obtained with TonoVet Plus and the lowest with Kowa HA-2. All tonometers accurately measured IOP in dogs, including the latest TonoVet Plus, which showed an excellent correlation coefficient.

## Introduction

Tonometers are an important tool for measuring intraocular pressure (IOP) for the diagnosis of eye diseases that can lead to irreversible blindness, including those that cause an increase in IOP, such as glaucoma, which can lead to optic neuropathy and is characterized by the death of retinal ganglion cells and their axons with accompanying vision loss; or those that reduce IOP, such as uveitis, which is usually due to secondary causes, such as infections, inflammation, trauma, or tumoral processes [1-6]. Early determination of an increase in IOP in glaucoma and a decrease in uveitis represents an important treatment success factor. It provides a more favorable prognosis for these diseases, as they are ophthalmopathies with great potential to induce irreversible blindness [7,8]. IOP measurement can be performed using manometry or tonometry. The most accurate method, considered as the “gold standard,” is direct or ocular manometry, which measures actual IOP in mmHg and consists of anterior chamber cannulation and measurement with a digital instrument or a mercury column [9-11]. Tonometers use different techniques to measure IOP and may be classified as contact, fixed, or portable [1,2,7]. In veterinary medicine, contact and portable tonometers are most commonly used, with the applanation method (Tono-Pen) [7,9], or more recently, the rebound method (TonoVet and TonoVet Plus) [12-14]. Other methods used are indentation (Schiötz) [7,9] and applanation tonometry using the Goldmann prism (Perkins and Kowa HA-2) [15-19].

Applanation tonometry is based on the principle that the force required to flatten a given area of a sphere is equal to the pressure within the sphere (Imbert-Fick Law) [7]. Goldmann (fixed to the slit lamp) was the first applanation tonometer used in human medicine. Portable applanation tonometers that facilitated the examination of bedridden patients and children were later developed and are represented by those using the Goldmann prism (Perkins and Kowa HA-2), as well as others with different methodologies such as the Draeger and MacKay-Marg, which are no longer sold, and the Tono-Pen, which is a portable digital applanation tonometer widely used in veterinary medicine [1,2,7,8,16-19]. Recently, the rebound method, wherein a light probe is used to make momentary corneal contact, has been introduced. The software analyzes the probe’s deceleration and contacts time as it touches the cornea. In simpler terms, the faster it slows down, the shorter the time that the probe contacts the cornea and the greater the IOP. The probe used is disposable, avoiding microbiological contamination, and the measurements are performed without topical anesthesia, making it a good alternative for ocular examination. Rebound tonometry is well tolerated and causes minimal stress and discomfort. It is marketed under the name of TonoVet; for laboratory animals, Tonolab, and more recently, TonoVet Plus was developed [12-15]. Because the cornea of most animals differs from the human cornea, human tonometer needs to be validated for use in animals. It is mandatory to perform direct postmortem manometry to calibrate the device using a calibration curve and equation of linear regression with various manometer versus tonometer IOP measurements. Further, validation by *in vivo* study of real IOP versus tonometer IOP measurements is also required [9,11,20]. Direct manometry versus tonometry has been studied in several species to validate, calibrate, or confirm the efficacy and accuracy of measuring IOP with various types of tonometers. In dogs, we can cite: Mackay-Marg, Tonair, and EMT-20 in 1977 [9], Mackay-Marg, Tono-Pen, and Challenger in 1990 [21], Mackay-Marg and Tono-Pen in 1992 [22], TonoVet in 2005 [13], Perkins in 2009 [16], and Kowa HA-2 in 2016 [18].

To date, there have been no studies in the literature that have compared the main tonometry methodologies used in veterinary medicine, such as rebound (TonoVet and TonoVet Plus) and applanation (Tono-Pen Avia Vet) using the Goldmann prism (Kowa HA-2) in dogs. Thus, this study aimed to evaluate and compare the accuracy of these tonometers with different methodologies for measuring IOP in dogs.

## Materials and Methods

### Ethical approval

This study was approved by the Ethical Committee on Animal Use of UNOESTE (Protocol No. 4177) and was conducted in accordance with the Association for Research in Vision and Ophthalmology for the use of animals in ophthalmic and visual research.

### Study period and location

This study was performed from February 2018 to February 2020 at the Veterinary Hospital of the UNOESTE, Presidente Prudente, SP, Brazil.

### Animals and study design

To determine the minimum sample size required to estimate the mean IOP measurements, we used the formula described by Pagano *et al*. [23], with a standard deviation based on the results obtained by Tofflerine *et al*. [24]. Based on these parameters, we concluded that a minimum of 130 eyes would be required for the present study. The actual sample size (152 eyes) used in this study was larger than the minimum to improve reliability. All eyes included in the study underwent slit-lamp examination (SL-15, Kowa, Tokyo, Japan) and indirect ophthalmoscopy (Pocket Jr, Welch Allyn, New York, USA) to rule out any ophthalmic conditions that affect the IOP or ocular surface.

A total of 152 eyes were used from 76 dogs, aged 1-10 years, weighing between 4 and 35 kg, divided into three groups: Postmortem study (20 healthy eyes of 10 dogs, animals with mortis caused by various reasons) at the Veterinary Hospital of the UNOESTE and authorized for autopsy examination (postmortem up to 24 h); *in vivo* study (20 healthy eyes of 10 healthy dogs from the University kennel, by normal laboratory, clinical and ophthalmic examinations), and outpatient study (112 eyes of 56 dogs) from routine ophthalmic care in the Veterinary Teaching Hospital of UNOESTE. Sixty-six healthy eyes from 33 healthy dogs underwent clinical and laboratory tests to confirm that they were healthy; 20 eyes from 10 dogs presented with clinical signs of glaucoma (congested episcleral vessels, blepharospasm, visual impairment, corneal edema, buphthalmia, fixed dilated pupil, anterior chamber changes, lens dislocation, retinal degeneration, and optic disk excavation), and 26 eyes from 13 dogs presented with clinical signs of uveitis (photophobia, blepharospasm, pain, epiphora, aqueous flare, keratic precipitates, hypopion, hyphema, ciliary injection, corneal edema, miosis, and anterior or posterior synechiae). All the owners of the animals signed an informed consent form before participating in the study.

**Figure-1:**
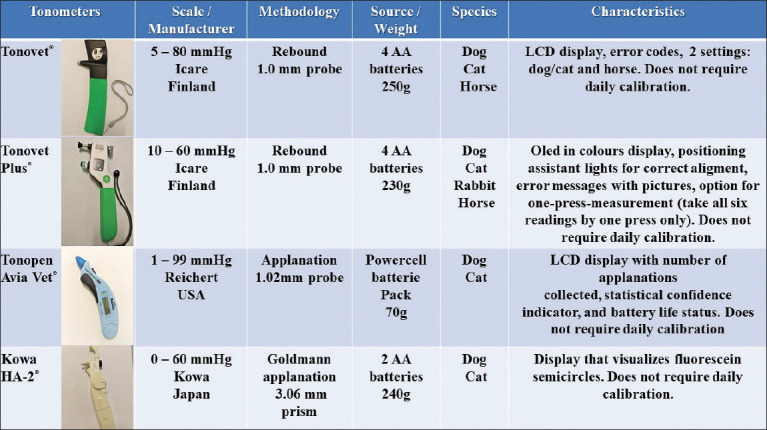
Main characteristics of TonoVet, TonoVet Plus, Tono-Pen Avia Vet, and Kowa HA-2 tonometers.

IOP measurements with the tonometers were obtained by the same examiner (JVGCP) for TonoVet (Icare, Vantaa, Finland), TonoVet Plus (Icare, Vantaa, Finland), and Tono-Pen Avia Vet (Reichert, New York, USA), and SFA for Kowa HA-2 (Kowa, Tokyo, Japan). The tonometers were used according to the manufacturer’s instructions, and the dog’s head was in a horizontal position in relation to the tonometers [15]. All probes were directed to the center of the cornea. Figure-1 presents the main characteristics of the tonometers. In addition, the main advantages and disadvantages of each tonometer were evaluated by the researchers involved in this study at the end of the experiment, analyzing the following requirements of each device: Accuracy, training required for adequate IOP measurements, need for topical anesthesia, probe (size, price, and safety), battery cost–benefit, and tonometer price.

### Postmortem study

To study the accuracy of the tonometers at different IOP values, a postmortem study was performed by comparing real IOP values among the tonometers using direct ocular manometry. The methodology was based on previously published studies [16-18]. The eyelids were separated with a blepharostat. The anterior chamber was cannulated with a 23-gauge scalp, 2 mm posterior to the lateral limb, for 10 h in the right eye and 2 h in the left eye. Cyanoacrylate glue was applied around the needle to prevent leakage of the aqueous humor. The needle was connected to a polyethylene tube connected to a three-way stopcock, connected through another polyethylene tube to a 0.9% saline reservoir on one side and an aneroid manometer (Missouri, São Paulo, Brazil) on the other side. The aneroid manometer was in a zero position relative to the center of the eye. The calibration curve for manometry versus tonometry was determined by artificially raising the IOP in 5 mmHg increments, up to 60 mmHg (10-60 mmHg). Three readings were taken at each IOP level using a tonometer, and the average was calculated. Following an order for tonometer use, starting with TonoVet, TonoVet Plus, Tono-Pen Avia Vet, and Kowa HA-2, 1% fluorescein eye drops were first instilled to form the fluorescein semicircle.

### *In vivo* study

To evaluate the accuracy of the tonometers, an *in vivo* study was performed to compare the actual IOP obtained through direct manometry measurements in anesthetized dogs, and that obtained with the tonometers. The animals were anesthetized using the following protocol: Pre-anesthetic medication with 0.2% acepromazine (Acepran, Vetnil, São Paulo, Brazil) at a dose of 0.05 mg/kg IV followed by induction with 10 mg/mL propofol (Propovan, Cristalia, São Paulo, Brazil) at a dose of 5 mg/kg IV and intubation through an endotracheal tube for anesthetic maintenance with 1.5% isoflurane (Isoflurano, Biochimica, Rio de Janeiro, Brazil). We used 10 mg/mL of the neuromuscular blocker atracurium besylate (Tracur, Cristalia, São Paulo, Brazil) at a dose of 0.1 mg/kg IV. To reverse the possible effects of atracurium besylate, 0.5 mg/mL neostigmine (Normastig, Union Química, São Paulo, Brazil) at a dose of 0.01-0.04 mg/kg plus 0.25 mg atropine sulfate (Pasmodex, Isofarma, São Paulo, Brazil) at a dose of 0.044 mg/kg was used. After the animals were anesthetized, the eyelids were separated with a blepharostat. Three IOP measurement readings were taken with the tonometers, and the mean was calculated, in the following sequence: TonoVet, TonoVet Plus, Tono-Pen Avia Vet, and Kowa HA-2 tonometers. Before the use of Tono-Pen Avia Vet, a topical anesthetic was used with one drop of 1% tetracaine hydrochloride + 0.1% phenylephrine hydrochloride-based eye drops (Anestésico, Allergan, São Paulo, SP, Brazil); before measurement with the Kowa HA-2 tonometer, a drop of 1% fluorescein eye drops (Fluoresceína, Allergan, São Paulo, SP, Brazil) was instilled for the formation of fluorescein semicircles. To prevent transmissible eye diseases, the protocol for each tonometer after use was as follows: TonoVet and TonoVet Plus probe change (Icare tonometer probe, Icare, Vantaa, Finland), Tono-Pen Avia Vet film change (Ocu-Film, Reichert, New York, USA), and Kowa HA-2 Goldmann prism (Kowa, Tokyo, Japan) were immersed in a 3% hydrogen peroxide solution, maintained for 5 min, then immersed in a 0.9% physiological solution and dried with a sterile gauze [25]. After tonometer IOP measurement, direct manometry was performed, as previously described in postmortem study. After the IOP measurement was obtained with the aneroid manometer, the needle was removed from the anterior chamber. Subsequently, cyanoacrylate glue was instilled using 25×7 needle at the puncture site to seal the perforation and prevent extravasation of the aqueous humor [26]. Following this procedure, the animals were treated with one drop 3×/day of tobramycin antibiotic eye drops (Tobrex, Novartis, Sao Paulo, Brazil) and diclofenac anti-inflammatory eye drops (Still, Allergan, São Paulo, Brazil), for 1 week, and assessed by daily ophthalmic examination.

### Outpatient IOP measurement study

To evaluate the routine clinical use of the tonometers, an outpatient IOP measurement study was performed in normal eyes of healthy dogs and eyes with clinical signs of glaucoma and uveitis. Tonometer readings were performed in the following order of use: TonoVet, TonoVet Plus, Tono-Pen Avia Vet after prior instillation of one drop of anesthetic eye drops, and Kowa HA-2 after instillation of one drop of 1% fluorescein eye drops for the formation of fluorescein semicircles. The previous studies reported that the interval between the uses of two tonometers could be 2 min [12] or 1 min [27]. In the present study, there was a 2 min interval between the uses of each tonometer.

### Statistical analysis

In the postmortem study, regression lines were constructed for the measured values of manometry versus tonometry, and the coefficient of determination (r^2^) and the linear regression equation was calculated. The Bland–Altman agreement analysis was used to compare the two methods of measuring IOP (manometry vs. tonometry). A series of agreements were defined as a mean bias of ± 2 standard deviations. In the in vivo and outpatient studies, the mean and standard deviation of the measured IOP values were calculated and compared statistically using analysis of variance. A significance level of 5% (p<0.05) was adopted.

## Results

**Figure-2:**
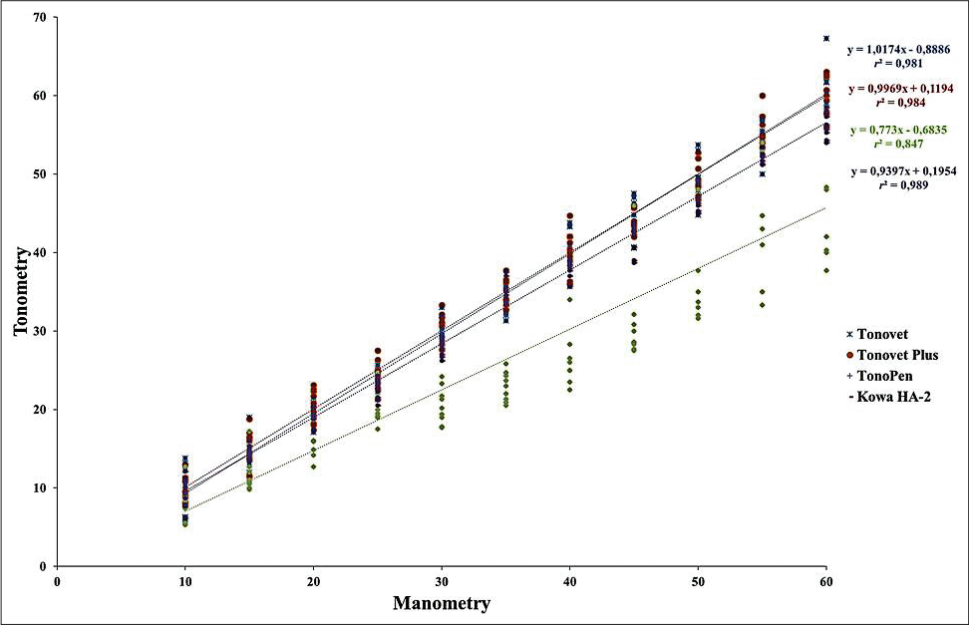
Comparison of intraocular pressure measurements in mmHg between manometry (aneroid manometer) versus tonometry (TonoVet, TonoVet Plus, Tono-Pen Avia Vet, and Kowa HA-2) in 10 dogs (n=20 eyes) in a postmortem study. The solid line is the calculated regression line. r^2^ (correlation coefficient).

**Figure-3:**
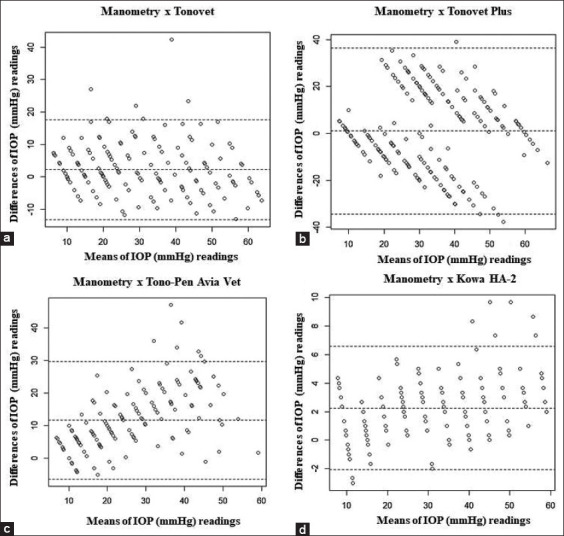
Bland–Altman plot comparing intraocular pressure in mmHg in dogs (n=20 eyes): (a) TonoVet tonometer and manometer, (b) TonoVet Plus tonometer and manometer, (c) Tono-Pen Avia Vet tonometer and manometer, and (d) tonometer Kowa HA-2 and manometer.

In the postmortem study, the tonometers, in terms of their correlation coefficient (r^2^) values (Figure-2) in descending order, were Kowa HA-2 (0.989), TonoVet Plus (0.984), TonoVet (0.981), and Tono-Pen Avia Vet (0.847). The linear regression equation was y=1.017x−0.8886 (TonoVet), y=0.9969x+0.1194 (TonoVet Plus), y=0.773x−0.6835 (Tono-Pen Avia Vet), and y=0.9397x+0.1954 (Kowa HA) -2). The Bland–Altman plot comparing the various tonometers with the manometry results are shown in Figure-3.

All IOP values (mean±standard deviation and minimum and maximum range) in the *in vivo* study are described in Table-1, and the outpatient study is described in Table-2. There was no significant difference in the *in vivo* study (p>0.05) between the IOP values measured using manometry and TonoVet, Tono-Pen Avia Vet, and Kowa HA-2 tonometers. TonoVet Plus was the only tonometer that presented significantly higher IOP values (p<0.05) than those obtained through manometry and using other tonometers. In the outpatient study, the only tonometer that showed a significant difference (p<0.05) between the measured IOP values and that obtained using other tonometers in all studied groups (healthy, and those with signs of glaucoma and uveitis) was TonoVet Plus. IOP values measured with TonoVet Plus averaged 3-5 mmHg higher than those measured with the other tonometers in all the groups studied.

**Table-1:** Means and standard deviations for the IOP values obtained in the *in vivo* study in 20 eyes from 10 dogs of manometry versus tonometry with TonoVet, TonoVet Plus, Tono-Pen Avia Vet, and Kowa HA-2 tonometers.

Animal	Manometer	TonoVet	TonoVet Plus	Tono-Pen Avia Vet	Kowa HA-2
1	15.5	22.0	22.0	18.3	18.0
2	15.5	16.0	22.0	15.8	15.3
3	19.0	20.5	21.3	15.5	18.2
4	20.0	22.7	24.0	19.5	17.0
5	14.0	14.0	17.2	17.0	13.5
6	14.5	18.0	21.2	14.0	13.5
7	16.5	18.0	21.8	22.7	16.8
8	15.5	16.5	17.0	15.7	16.2
9	16.0	15.0	18.3	15.5	15.3
10	21.5	18.5	21.3	16.8	16.8
Mean±SD*	16.8±2.5**^a^	18.1±2.9^a^	20.6±2.3^b^	17.1±2.5^a^	16.1±1.7^a^
Range	14.0-21.5	14.0-22.7	17.0-24.0	14.0-22,7	13.5-18.2

* Mean±standard deviation.

** Different superscript letters indicate significant differences (p<0.05). IOP=Intraocular pressure

**Table-2:** Means and standard deviations of IOP values obtained with TonoVet, TonoVet Plus, Tono-Pen Avia, and Kowa HA-2 tonometers in the outpatient study of 112 eyes from 56 dogs (66 healthy eyes, 20 eyes with glaucoma, and 26 eyes with uveitis) in dogs treated at the ophthalmology department of UNOESTE Veterinary Hospital, Presidente Prudente, SP, Brazil.

Group	TonoVet	TonoVet Plus	Tono-Pen Avia Vet	Kowa HA-2
Health				
Mean±SD*	16.8±3.2^a^	19.2±2.9^b^	16.2±2.4^a^	15.0±1.3^a^
Range	11.7-24.5	14.7-25.0	13.3-23.0	12.5-18.0
Glaucoma				
Mean±SD*	30.2±3.5^a^	35.0±6.1^b^	29.5±4.2^a^	23.9±5.0^a^
Range	26.0-34.6	29.5-44.8	26.0-37.7	20.0-33.7
Uveitis				
Mean±SD*	14.2±1.4^a^	17.6±1.9^b^	13.7±2.1^a^	12.6±1.7^a^
Range	12.0-15.5	15.5-20.5	11.0-16.5	10.0-14.8

* Mean±standard deviation.

**Different superscript letters indicate significant differences (p<0.05). IOP=Intraocular pressure, SD=Standard deviation

## Discussion

This is the first study to compare the main tonometers used in dogs, an excellent animal model for the study of tonometer efficacy, to measure IOP with different types of methodology: Rebound (TonoVet and TonoVet Plus) and applanation (Tono-Pen Avia Vet), with the applanation methodology of Goldmann (Kowa HA-2). According to the authors, TonoVet and TonoVet Plus generally have more advantages in their daily use, but all tonometers showed excellent accuracy for IOP measurement in dogs (Table-3). The IOP values that were closest to the IOP values measured by manometry (*in vivo* study) were, in descending order, Kowa HA-2, Tono-Pen Avia Vet, and TonoVet; TonoVet Plus differed statistically (p>0.05) with higher values than with manometry (Table-1).

**Table-3:** Characteristics positive (+) or negative (−) in the various parameters observed by the authors in this study in dogs using TonoVet, TonoVet Plus, Tono-Pen Avia Vet, and Kowa HA-2 tonometers.

Characteristics	Tonometers			

	TonoVet	TonoVet plus	Tono-Pen Avia Vet	Kowa HA-2
Accuracy	+	+	+	+
Training (facility)	+	+	+	−
Anesthesia (without)	+	+	−	−
Probe (smaller size)	+	+	−	−
Probe (disposable)	+	+	+	−
Battery (cost/benefit)	+	+	−	+
Price (smaller)	−	−	−	+

In the outpatient study (Table-2), measuring IOP in healthy eyes with clinical signs of glaucoma and uveitis, TonoVet Plus yielded significantly higher IOP values (p<0.05) than other tonometers. In both the *in vivo* and outpatient studies, the IOP values measured by TonoVet Plus averaged 3-5 mmHg higher than those measured with the other tonometers, which is consistent with some recent studies, such as the study by Ben-Shlomo and Muirhead [28], who compared IOP values in healthy dogs, obtained with TonoVet (15.0±3.2 mmHg; range 7-22 mmHg), TonoVet Plus (19.2±3.1 mmHg; range 11-25 mmHg), and Tono-Pen Avia Vet (12.8±2.9 mmHg; range 6-19), and concluded that TonoVet Plus indicated significantly higher IOP values than TonoVet and Tono-Pen Avia Vet. These results were in agreement with those presented by Guresh *et al*. [29], who conclude TonoVet Plus produced consistently and significantly higher IOP readings. Nevertheless, the measurements did not exceed the expected IOP range in normal dogs.

The mean IOP values measured with all tonometers in dogs with glaucoma were greater than the mean measured value in healthy eyes. The mean IOP value in dogs with uveitis was less than the mean measured value in dogs with healthy eyes. The IOP values above the normal range are compatible with glaucoma. In contrast, IOP values below this range are compatible with uveitis, which is the trend that was observed in this study with all tonometers [3,6].

In another recent study [30] comparing the Schiotz tonometer with the Tono-Pen Avia Vet tonometer, the IOP values with the Schiotz tonometer were in the range of 12-24 mmHg, with an average of 16.3±2.1 mmHg. Those with the Tono-Pen Avia Vet tonometer were in the range of 11-25 mmHg, with an average of 18.1±3.8 mmHg. The results between the two tonometers differed significantly by 1.79 mmHg. In our study, the average IOP with Tono-Pen Avia Vet was slightly low (16.2±2.4 mmHg), and the variation was quite similar, from 13 to 23 mmHg. The correlation coefficient found with Tono-Pen Avia Vet in the previous study was 0.894, close to the correlation coefficient found in our study (0.847).

Nagata *et al*. [31] compared the TonoVet and Tono-Pen XL tonometers for the measurement of IOP in dogs. They found that the values measured by TonoVet were underestimated, compared to those measured by Tono-Pen XL, when the measurements were 5-15 mmHg. The inverse occurred when the pressure was above 25 mmHg, when Tono-Pen XL yielded values below those reported using TonoVet. This was in contrast to our study comparing TonoVet with Tono-Pen Avia Vet, as, throughout the study, Tono-Pen Avia Vet values were lower than those obtained using TonoVet. Kulualp *et al*. [32] also compared TonoVet and Tono-Pen Vet in clinically normal Turkish Shepherd dogs. The IOP measurements were very close to those found in our study, with 17.63±3.34 mmHg and 16.8±3.2 mmHg measured with TonoVet and 14.95±2.92 mmHg and 16.2±2.4 mmHg measured with Tono-Pen Vet.

The correlation coefficient (r^2^) observed in our study with the Kowa HA-2 tonometer was 0.989. The average value was 14.2±1.6 for the measurements of healthy eyes, 23.9±5.0 for eyes with glaucoma, and 12.8±1.9 for eyes with uveitis. These results were very close to the values reported by Andrade *et al*. [18], with a correlation coefficient of 0.993, 15.1±1.8 for healthy eyes, 25.2±4.0 for eyes with glaucoma, and 10.1±2.3 for eyes with uveitis.

The Bland–Altman plot shows that all tonometers underestimated the IOP values relative to manometry in high IOPs (30-60 mmHg). This is in agreement with the study by Minella *et al*. [33], who validated TonoVet Plus and Tono-Pen Avia Vet in normal canine eyes and observed this trend at 30-70 mmHg values.

## Conclusion

This is the first study to draw a comparison between Goldman’s methodology with the Kowa HA-2 tonometer and other type tonometers, rebound (TonoVet and TonoVet Plus) or applanation (Tono-Pen Avia Vet) in dogs. There was a strong correlation between IOP values obtained with manometry and those obtained using TonoVet, TonoVet Plus, Tono-Pen Avia Vet, and Kowa HA-2, demonstrating the high accuracy of all the tonometers. The highest IOP values were measured with TonoVet Plus and the lowest with Kowa HA-2, reinforcing the need for a differentiated IOP value table for each type of tonometer. All tonometers accurately measured IOP in dogs, including the latest TonoVet Plus, which showed an excellent correlation coefficient.

## Authors’ Contributions

JVGCP: Designed and conducted the experiment, performed all animal examinations and tests, analyzed data, prepared the graphs, figures, and tables, and prepared the manuscript. FFN, GJGE, CLR, and GPK: Performed all animal examinations, and tests analyzed the data, and prepared the manuscript. RG: Designed and analyzed the data, prepared the graphs, figures, and tables, and prepared the manuscript. SFA: Conceptualized the aim of the study, planned, supervised, designed, and conducted the experiment, performed all animal examinations and tests, analyzed data, prepared the graphs, figures, and tables, and prepared the manuscript. All authors read and approved the final manuscript.
